# Transcriptomic analysis of *Blumea laciniata* responses to PEG-simulated drought stress

**DOI:** 10.3389/fpls.2025.1695003

**Published:** 2025-12-18

**Authors:** Hongjuan Wang, Yongdong Dai, Mingyuan Zhang, Yang Chen

**Affiliations:** 1 Biotechnology Research Institute, Chongqing Academy of Agricultural Sciences, Chongqing, China; 2 Chongqing Key Laboratory of Adversity Agriculture Research, Chongqing, China; 3 Key Laboratory of Evaluation and Utilization for Special Crops Germplasm Resource in the Southwest Mountains, Ministry of Agriculture and Rural Affairs, Chongqing, China

**Keywords:** *B. laciniata*, drought stress, RNA-seq, WGCNA, TFS

## Abstract

**Introduction:**

*Blumea laciniata* (*B. laciniata*), a well-documented ethnomedicinal species in Chinese pharmacopeia, has demonstrated significant therapeutic efficacy against various infectious diseases. With the growing number of studies, medicinal plants are being acknowledged as valuable natural resources for combating stress. Elucidating the drought tolerance mechanisms of such species is crucial for formulating adaptive cultivation strategies to mitigate climate change-induced agricultural challenges.

**Methods:**

In this study, drought stress was induced using two polyethylene glycol (PEG) concentrations (20% and 30%). Furthermore, RNA-seq and WGCNA (Weighted gene co-expression network analysis) were conducted on *B. laciniata* plants at five time points (0, 1, 2, 4, and 7 days) pre- and post-stress exposure.

**Results:**

*B. laciniata* demonstrates natural drought tolerance, as observed in field studies. PEG-induced drought stress at two concentrations (20% and 30%) impaired leaf development, resulting in chlorosis, curling, wilting, and necrosis, with 30% PEG showing greater damage. Moreover, pro and SOD levels increased with stress duration. RNA-seq analysis demonstrated distinct transcriptional reprogramming in *B. laciniata* leaves under PEG stress. Venn and KEGG enrichment analyses revealed that the plant primarily responds to drought stress by regulating phenylpropanoid and flavonoid biosynthesis pathways. Furthermore, WGCNA analysis identified two transcription factors (TFs), GRF2 and NF-YA3, as key regulators associated with the drought resistance of *B. laciniata*.

**Discussion:**

Our study provides a theoretical basis for elucidating the molecular mechanisms underlying drought resistance in *B. laciniata* plants and provides new genetic resources for the study of drought resistance in this crop.

## Introduction

Drought, as a predominant abiotic stress, exerts multifaceted impacts on crops by disrupting morphological traits, physiological functions, and biochemical pathways, thereby constraining growth progression, developmental stages, yield potential, and end-product attributes (Lesk et al., 2016, Gupta et al., 2020). Climate models project a significant escalation in both the severity and recurrence of drought episodes under anticipated future climatic conditions (Easterling et al., 2000). In China, drought-induced annual agricultural production losses are estimated at approximately 4.2 billion USD, accounting for 0.23% of the national GDP (Su et al., 2018). Consequently, the screening and identification of key drought-resistant genes, coupled with elucidating the adaptive mechanisms of plants to drought stress, are pivotal for achieving agricultural sustainability (Raposo et al., 2023). Plants face drought-induced water, oxidative, and nutrient stresses, leading to adaptations like adjusting leaf wax, epidermal hair, and stomata, changing roots, and balancing hormones and antioxidants (Liu et al., 2023; Ilyas et al., 2021; Martin-StPaul et al., 2017). Plants initiate *de novo* biosynthesis of compatible solutes to achieve osmotic homeostasis under soil water deficit stress. These acclimation responses are mediated through the coordinated expression of multiple genes within an intricate regulatory network (Zhu and JK, 2016).

TFs serve as pivotal components within signal transduction cascades, integrating stress signal perception with the transcriptional activation of stress-responsive genes (Dhriti and Ashverya, 2015; Golldack et al., 2014; Hrmova and Hussain, 2021; Manna et al., 2021; Tang and Xia, 2025). As multifunctional regulatory proteins, TFs exhibit the capacity to coordinately modulate multiple stress-adaptive pathways in plants, thereby constituting versatile molecular tools for engineering both regulatory networks and stress response mechanisms (Hrmova and Hussain, 2021; Manna et al., 2021; Tang and Xia, 2025). Recently, the relationships between the structure and function of many plant TFs related to drought and related stresses have been identified, leading to the development of practical strategies for engineering plants with improved stress tolerance (Hrmova and Hussain, 2021; Manna et al., 2021; Tang and Xia, 2025). For example, *DREB2A* expression is transcriptionally suppressed through the specific binding of AtGRF7 to its cognate cis-regulatory motif ‘TGTCAGG’, a conserved DNA sequence element implicated in stress-responsive gene regulation (Kim et al., 2012). Under drought and salt stress, miR396 undergoes transcriptional induction, subsequently mediating the dissociation of DREB2A from its repressor AtGRF7, thereby enabling the activation of downstream stress-responsive gene networks (Kim et al., 2012). Under drought stress, CsMPK4a-mediated phosphorylation of CsWD40 exerts negative regulatory control over the flavonoid biosynthesis pathway in tea plants (Li et al., 2024b). Heterologous expression of *SbNF-YA6* in Arabidopsis significantly enhanced osmotic stress tolerance under mannitol-induced drought stress (Wu et al., 2025). In rice, the R2R3-MYB transcription factor OsFLP orchestrates drought adaptation through transcriptional modulation of *OsNAC1* and *OsNAC6* (Qu et al., 2022). Moreover, OsbZIP62 plays a role in ABA signaling pathways and enhances rice drought tolerance by controlling the expression of stress-related genes (Yang et al., 2019).

The advancement of high-throughput sequencing platforms and computational biology tools has facilitated the systematic integration of multi-omics approaches into deciphering molecular networks underlying plant drought adaptation strategies (Chen et al., 2024b; Jiang et al., 2021; Wang et al., 2025; Zhang et al., 2025). The application of genome-wide transcriptomic profiling methodologies enables comprehensive elucidation of plant gene regulatory architectures and facilitates systematic discovery of molecular components involved in abiotic stress adaptation pathways, particularly through high-resolution identification of differentially expressed stress-associated genes (Abdel-Ghany et al., 2020; Ye et al., 2025). These drought-responsive genetic elements represent promising candidates for improving plant drought resilience through modern breeding strategies and targeted genetic engineering interventions. Extensive transcriptomic investigations across diverse plant species have elucidated numerous drought-inducible genes (Namuunaa et al., 2025; Akter et al., 2025; Ost et al., 2023; Abdel-Ghany et al., 2020; Huang et al., 2014; Shinozaki et al., 2003; Taheri et al., 2022), whose encoded proteins participate in critical stress adaptation mechanisms. For example, two rice genes (*OsJAZ1* and *OsJAZ7*) involved in the JA signaling pathway were significantly up-regulated in H471 vs. HHZ under drought stress (Huang et al., 2014). *HvS40* and *HvA1* were also significantly induced by drought stress in barley (Ost et al., 2023). Transcriptome sequencing of drought-stressed and re-watered tomato leaves identified 966 mostly down-regulated differentially expressed genes (DEGs) under drought stress, including several genes encoding heat-shock proteins, cell wall-related enzymes as well as histones (Taheri et al., 2022). Transcriptome-wide analyses further reveal drought-induced reprogramming of gene expression networks across multiple metabolic pathways (Bashir et al., 2019). Plants rely on the enhancement of secondary metabolites to adapt and evolve to environmental shifts when faced with different stressors (Ahmed et al., 2021). Phenylpropanoid biosynthesis pathway plays an important role in plant response to abiotic stress, including drought stress (Vogt, 2010; Sun et al., 2025; Chen et al., 2024a). Drought stress also promoted the accumulation of flavonoid metabolites in many plant species (Ahmed et al., 2021; Feng et al., 2024; Li et al., 2024a).

*B. laciniata* has long been employed as a traditional herbal medicine for treating human infectious diseases in China (Li et al., 2004). Medicinal plants are known for their high secondary metabolite production. Drought stress reduces plant biomass, especially critical for medicinal plants, due to its impact on physiological and biochemical processes, which severely limits secondary metabolite production (Selmar et al., 2017). Growing research shows that medicinal plants are important resources for stress resistance (Ye et al., 2025; Vaghela and Gohel, 2023; Tan and Gören, 2024). The molecular mechanisms behind drought stress are still mostly unclear. Consequently, this study employed RNA-seq to produce an extensive gene expression profile related to the growth and development of *B. laciniata*, and to investigate the transcriptional responses in leaves triggered by drought. The objective is to elucidate the regulatory network of plant response to drought stress, offering essential data to enhance agricultural productivity.

## Materials and methods

### Plant materials and drought treatment

Seeds for this research were gathered from Jiulongpo district in Chongqing, China. They were germinated under controlled conditions in growth chambers and later grown in a greenhouse with a 16-hour light and 8 hours dark. Seeds that exhibited consistent growth were chosen and evenly transplanted into the hydroponic pots. Once they reached the trifoliate stage, PEG stress treatments were administered using two concentrations: 20% and 30% PEG6000. Samples of true leaves from *B. laciniata* were collected at intervals of 0, 1, 2, 4, and 7 days post-PEG treatment, with three duplicates for each time. These were rapidly frozen using liquid nitrogen and stored temporarily at -80°C.

### Proline content

Pro content was measured spectrophotometrically according to previous study (Bates et al., 1973), with slight modifications. 0.5 g of leaves in total were homogenized in 3% aqueous sulfosalicylic acid. After centrifuging the samples at 12000 rpm for 10 minutes, 2 ml of the supernatant was transferred to a test tube. An equal volume of acid ninhydrin and glacial acetic acid was added to the supernatant, and the mixture was boiled in a water bath for 45 minutes. Once the color developed, the samples were cooled on ice entirely before adding equal amounts of toluene and mixed thoroughly until a distinct layer formed on the solution’s surface. The toluene layer, isolated from the water phase, was gathered, and the optical density of the samples was measured at 520 nm using a spectrotometer Helios^®^ Epsilon visible 8nm bandwidth (Thermo Fisher Scientific, USA). The amount of Pro was estimated using the Pro standard curve and was expressed as μg/g FW.

### Superoxide dismutase activity

SOD activity was determined by the nitrogen blue tetrazolium (NBT) method (Beauchamp and Fridovich, 1971). For enzymatic extraction, 0.1 g of tissue was added to a mixed solution consisting of 2 mL of 0.1 M sodium phosphate buffer (pH 7.0) with 1 mM EDTA, 1% PVP, and 0.01 M NaCl. The 3 mL reaction mixture included 0.1 M sodium phosphate buffer at pH 7.0, 13 mM methionine, 75 µM NBT, 20 µL enzyme extract, and 2 µM riboflavin. By adding riboflavin, the reaction was initiated. The mixture underwent 15 minutes of exposure to UV light at 15 W, and absorbance was measured at 560 nm.

### RNA-seq

Total RNA was extracted from the samples with the RNA Extraction kit (TIANGEN, Beijing, China). Agarose gel electrophoresis and the NanoDrop2000 spectrophotometer (NanoDrop Technologies, Wilmington, DE, USA) were used to evaluate the RNA quality and concentration. Quality testing, library construction, and sequencing for each sample were done at Norminkoda Biotechnology Co., Ltd (https://www.bionmkd.com/). Trinity (v2.6.5) was used for *de novo* transcriptome assembly to obtain a reference genome (Grabherr et al., 2011). The clean reads were localized using HISAT2 (2.2.1) to obtain unigenes (Kim et al., 2015). Gene expression values were determined using the fragments per kilobase of exon model per million mapped fragments (FPKM) analyzed by StringTie for all genes (Pertea et al., 2015). Raw counts served as the input for DESeq2 (v1.28.1) to detect differentially expressed genes (DEGs) (Love et al., 2014), with a Q value ≤ 0.05 and |log2fold change|>1 serving as the screening criteria for identifying DEGs according to differential expression. Gene annotation was performed by blasting the assembled transcripts against the NCBI (National Center for Biotechnology Information) non-redundant protein database (Nr), Swiss-Prot, GO, KOG, eggNOG, Pfam and KEGG databases with an E-value cutoff of 1e-5, and functional terms were assigned based on the best hits.

### Weighted gene co-expression network analysis

The WGCNA package in R was employed to perform co-expression analysis on all expression profiles with 27 samples using unsigned TOM similarity (Langfelder and Horvath, 2008). A sample cluster tree was created to eliminate outlier samples. The soft-threshold parameter was set to β = 12, achieving a scale-free topology fit R² > 0.8. Modules were required to have at least 200 genes, and a merge cut height of 0.25 was applied for merging. To identify the hub gene of each module, GS (gene significance).abs needed to be greater than 0.6 and MM (module membership).abs greater than 0.8. The coexpression network was visualized using Cytoscape software (version 3.10.3) (Shannon et al., 2003).

### Quantitative RT-PCR analysis

The EASYspin Plant RNA Extraction Kit (Aidlab, Beijing, China) was used to extract the total RNA. The PrimeScript™ RT Reagent Kit (TaKaRa, Kusatsu, Shiga, Japan) was then used to synthesize the first-strand cDNA from 1 µg of RNA. Following the manufacturer’s instructions, quantitative PCR was performed on a CFX96™ Real-Time System (Bio-Rad, Hercules, California, USA) using the 1× iQ™ SYBR Green Supermix (Bio-Rad, Hercules, California, USA), and the data were analyzed. The thermal cycling protocol consisted of a pretreatment (94°C, 3 min) followed by 40 amplification cycles (94°C, 30 s; 56°C, 30 s; and 72°C, 30 s). Three individual runs, serving as biological replicates, confirmed each test, and data from one of these replicates was employed to generate the expression chart. The actin gene (g16517) functioned as the reference gene. Gene-specific primers used for qRT-PCR are listed in **Supplementary Table S7**.

### Statistical analysis

RStudio (v2023.06.1–524) was used to perform principal component analysis (PCA) with the FactoMineR and factoextra packages (Lê et al., 2008). A column chart was created using GraphPad Prism 5 (v5.01). Bidirectional grouping bar chart plot analysis results were produced via the CNSknowall platform (https://cnsknowall.com, a comprehensive web service for data analysis and visualization, accessed on 22 July 2025).Venn diagrams were examined using the SRplot online data analysis and visualization service (https://www.bioinformatics.com.cn, accessed on 25 July 2025). Kyoto Encyclopedia of Genes and Genomes (KEGG) pathway enrichment analysis were applied using TBtools software (v2.3.40) (Chen et al., 2020).

## Results

### Effects of drought stress on *B. laciniata*

**Figure 1:**
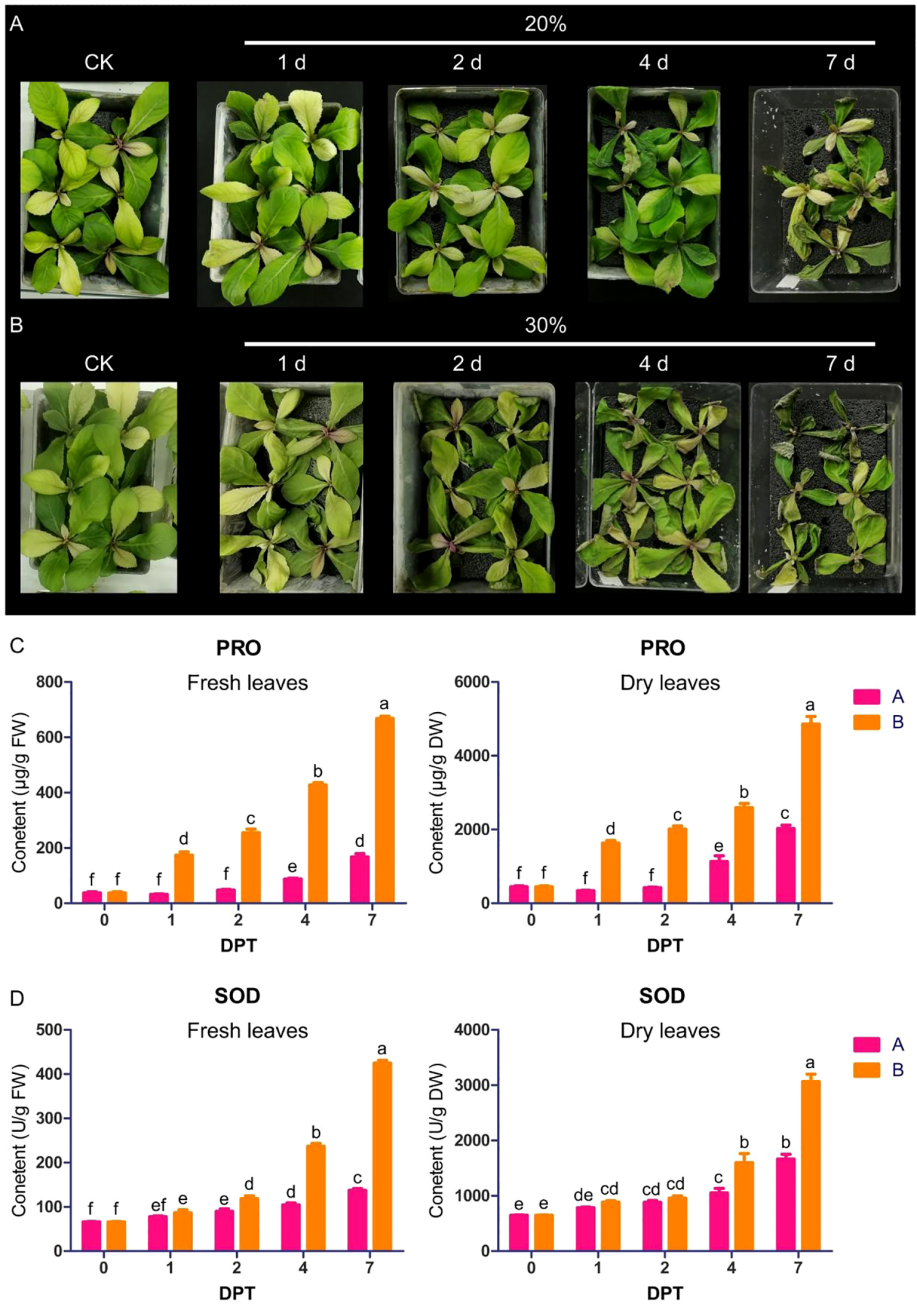
Phenotypic and physiological characteristics of *B. laciniata* leaves under drought stress. Phenotypic characteristics of *B. laciniata* leaves exposed to 20% PEG **(A)** and 30% PEG **(B)**. **(C)** Pro content in fresh and dry leaves, respectively. **(D)** SOD activity in fresh and dry leaves, respectively. FW: fresh weight. DW: dry weight. Three independent replicates were performed, with at least 3 plants being measured per replicate. Figures were drew with mean ± SD. Distinct letters signify *p* < 0.05, and identical letters signify *p* > 0.05.

Initial investigations confirmed the pronounced drought tolerance exhibited by *B. laciniata* (**Supplementary Figure S1**), prompting seed collection and propagation to establish experimental populations for systematic drought resistance evaluation. The drought resistance experiment employed two osmotic stress levels (20% and 30% PEG) to establish water deficit stress. Progressive phenotypic deterioration was observed in plants under drought stress, manifesting as sequential leaf yellowing, marginal curling/wilting, and scattered necrotic lesions, with 30% PEG treatment exhibiting exacerbated symptoms compared to 20% (**Figures 1A, B**). Physiological analyses revealed time-dependent accumulation of pro and SOD levels in *B. laciniata* leaves under both PEG concentrations, with significantly elevated levels under 30% PEG treatment relative to 20%, concordant with the phenotypic severity gradient (**Figures 1C, D**).

### An overview of transcriptomic

**Figure 2:**
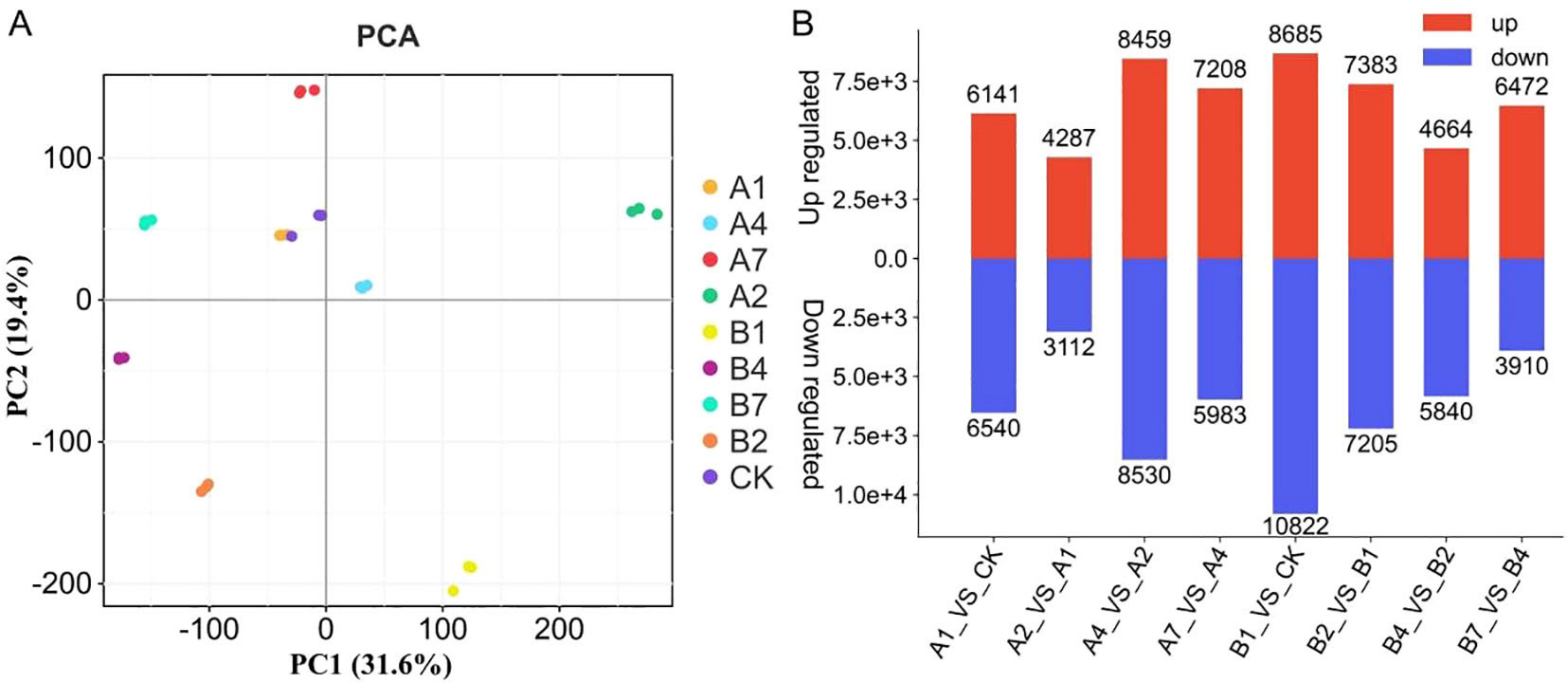
Overview of the transcriptomic profile of *B. laciniata* leaves treated with two PEG concentrations at six time points. **(A)** PCA score plots of genes identified in the two treatments at six time points based on FPKM values. **(B)** Overview of DEGs between samples at different time points. **(A, B)** represent the seedlings of *B. laciniata* treated with 20% and 30% PEG, respectively. CK represents 0 day post-PEG treatment. 1, 2, 4, 7 represent the days after PEG treatment.

To elucidate drought-responsive gene expression patterns in *B. laciniata*, leaf samples were harvested at six sequential time intervals spanning pre- and post-PEG treatment phases for comparative transcriptomic profiling. Transcriptomic profiling of *B. laciniata* under progressive drought stress yielded 1.4 billion clean reads from nine biological replicates sampled across five critical time points (0, 1, 2, 4, and 7 days post-PEG induction), enabling comprehensive identification of 57,502 expressed genes (**Supplementary Table S1**). PCA analysis was applied to transcriptome data of 27 samples to determine the associated variation. The analysis revealed that the first two principal components explained 51% of the variation, with PC1 and PC2 accounting for 31.6% and 19%, respectively (**Figure 2A**). The PCA results showed that the biological replicates were clustered together, indicating that the transcriptome data were reliable and repeatable (**Figure 2A**). To explore the transcriptional regulation in *B. laciniata* plants under drought stress, DEGs were analyzed using 27 samples. A total of 39,215 DEGs in the eight comparisons (A1 vs. CK, A2 vs. A1, A4 vs. A2, A7 vs. A4, B1vs. CK, B2 vs. B1, B4 vs. B2, and B7 vs. B4), constituting 68.2% of the genome, were identified (**Supplementary Table S2**). Comparative transcriptomic analysis of *B. laciniata* under drought stress revealed significant differential gene expression (DEG) dynamics across eight experimental comparisons (range: 7,399–19,507 DEGs). The A2 vs. A1 contrast exhibited minimal responsiveness (7,399 DEGs; 58% upregulated, 42% downregulated), whereas B1 vs. CK displayed maximal responsiveness (19,507 DEGs; 45% upregulated, 55% downregulated) (**Figure 2B**). These findings underscore drought-induced transcriptional plasticity in seedling leaves.

### DEG analysis of transcriptomic data

**Figure 3:**
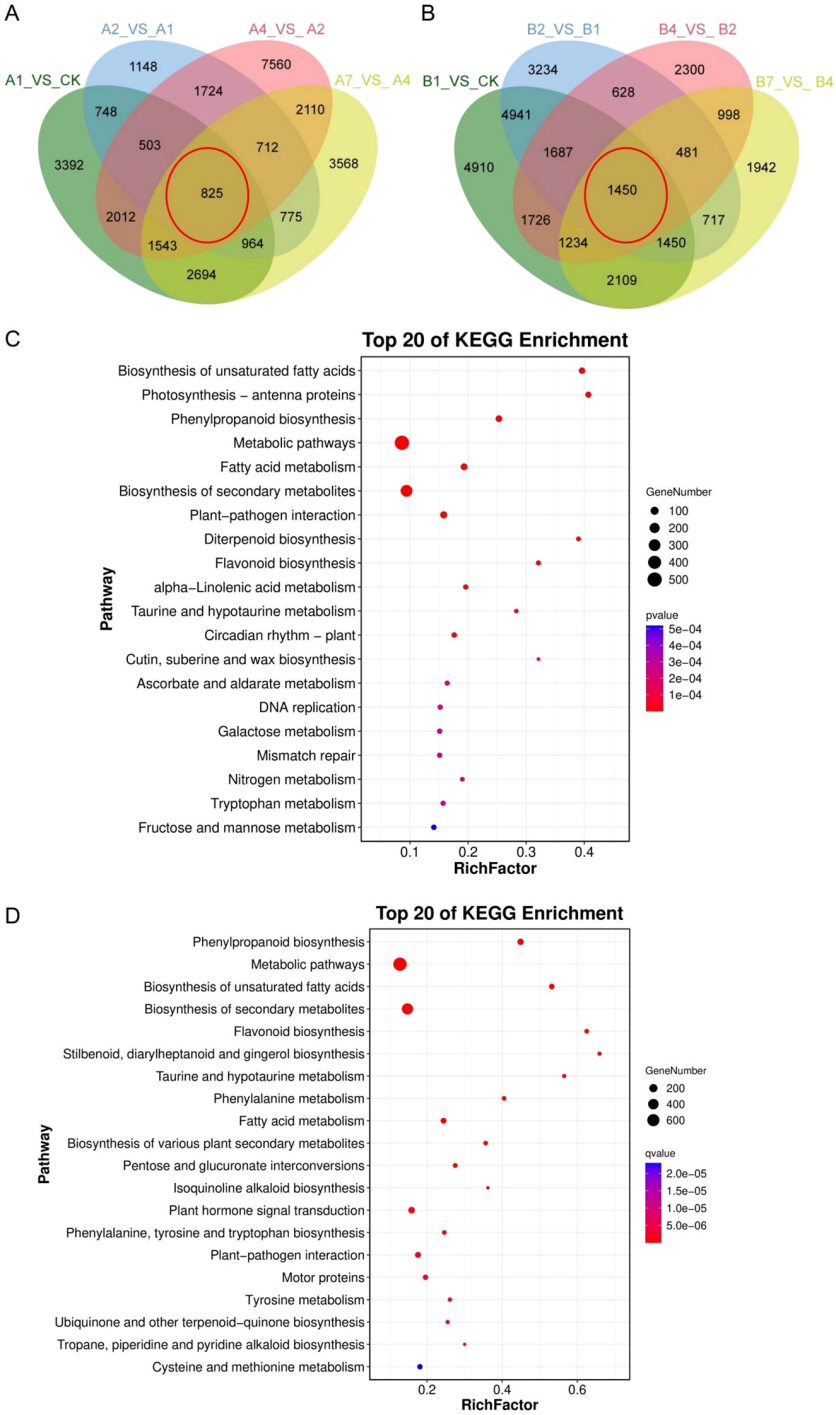
Venn and KEGG enrichment analysis DEGs across comparison groups. **(A, B)** Venn diagrams depicting DEG counts across comparison groups, with each red line indicating the intersections among the four comparison groups. The control group displayed on the right side of each comparison. **(C, D)** The rich factor plot of the KEGG pathway enrichment analysis results. The ordinate represents the name of the pathway, the size of the dot represents the number of genes, and the color represents the Q value (Q value < 0.05).

We conducted a Venn analysis to further characterize DEGs. The comparisons of A1 vs. CK, A2 vs. A1, A4 vs. A2, and A7 vs. A4 were examined, revealing 825 common genes that were differentially expressed across these comparisons (**Figure 3A**). Further KEGG enrichment analyses were conducted to examine the role of DEGs during drought stress. A total of 825 unigenes were linked to 125 pathways based on KEGG annotation, with 27 pathways showing significance at *p* < 0.01 and Q-value < 0.05 (**Supplementary Table S3**). The top 20 KEGG pathways based on the rich factor among the comparisons were screened out (**Figure 3C**). The top 20 KEGG pathways were identified based on the rich factor among the comparisons. The most significantly enriched pathways in the DEGs across different comparison groups are primarily associated with metabolism, including phenylpropanoid biosynthesis, flavonoid biosynthesis, cutin, suberine and wax biosynthesis, fatty acid metabolism, fructose and mannose metabolism, diterpenoid biosynthesis, and other pathways (**Figure 3C**).

The analyses also included the comparisons of B1 vs. CK, B2 vs. B1, B4 vs. B2, and B7 vs. B4. A total of 1450 common genes showed differential expression across these four comparisons (**Figure 3B**). The DEGs identified were further examined for their related KEGG pathways. A total of 1450 unigenes were linked to 124 pathways based on KEGG annotation, with 37 pathways showing significance at *p* < 0.01 and Q-value < 0.05 (**Supplementary Table S3**). The top 20 KEGG pathways were identified based on the rich factor among the comparisons (**Figure 3D**). The pathways most significantly enriched in the DEGs across different comparison groups are primarily associated with metabolism, such as phenylpropanoid biosynthesis flavonoid biosynthesis, stilbenoid, diarylheptanoid and gingerol biosynthesis, fatty acid metabolism, cysteine and methionine metabolism, and other pathways (**Figure 3D**). Notably, both phenylpropanoid and flavonoid biosynthesis were enriched in the two intersections, as it suggests these processes may significantly contribute to the drought tolerance of *B. laciniata*.

### Identification of the hub module and hub gene in WGCNA network

**Figure 4:**
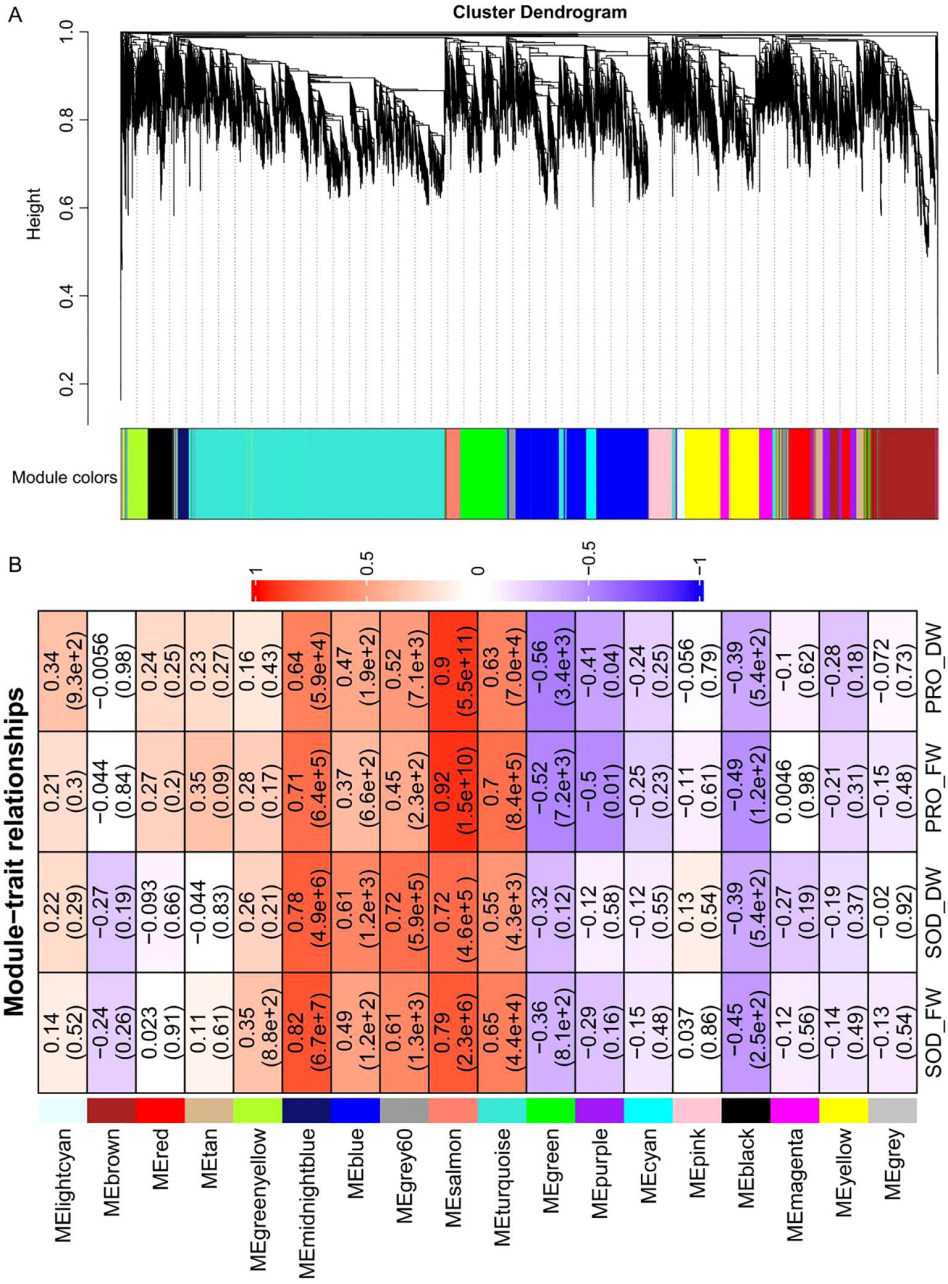
WGCNA analysis. **(A)** Hierarchical cluster dendrogram constructed by WGCNA, on which each leaf represents a gene. 18 merged modules (based on a threshold of 0.20) identified by weighted-gene co-expression network. Branches with different colors correspond to 18 different modules. **(B)** Pearson correlation coefficient and *p* values for significant pro or SOD associated WGCNA modules. The GS value for each module–trait pair is shown by color intensity and the number in the upper position of the box, *p* values is represented by the numbers in the brackets below in the box.

To identify DEGs and select the best unigenes under drought stress, all genes of 27 samples were utilized to build the WGCNA network. The genes within the same module exhibited similar expression patterns and were connected through average linkage clustering. A soft thresholding power of 12 was applied to maintain a scale-free network (**Supplementary Figure S2**). Modules with a height cut-off of 0.25 were combined, resulting in the identification of 18 modules (**Figure 4A**). Genes that did not belong to any module, totaling 77, were placed in the gray module. This gray module was determined to be non-co-expressed and will be excluded from further analysis. The turquoise module, with 8701 genes, had the most genes, while the grey60 module, with 236 genes, had the fewest (**Supplementary Table S4**).

Additionally, the connection between the modules and traits was assessed to pinpoint the hub module. Correlation coefficients between module content and traits varied widely, ranging from -0.56 to 0.92 (**Figure 4B**). Notably, the midnightblue module, which includes 375 genes, showed GS values over 0.78 between module content and SOD activity, highlighting a strong correlation between the genes in this module and SOD activity. The salmon module, which includes 421 genes, showed GS values over 0.9 between module content and pro, suggesting a strong link between these genes and pro (**Figure 4B**). Finally, 472 hub genes were identified in the midnightblue and salmon modules based on the criteria of |MM| > 0.8 and |GS| > 0.6 (**Supplementary Table S5**).

### Network analysis and validation candidate TFs associated with drought stress

**Figure 5:**
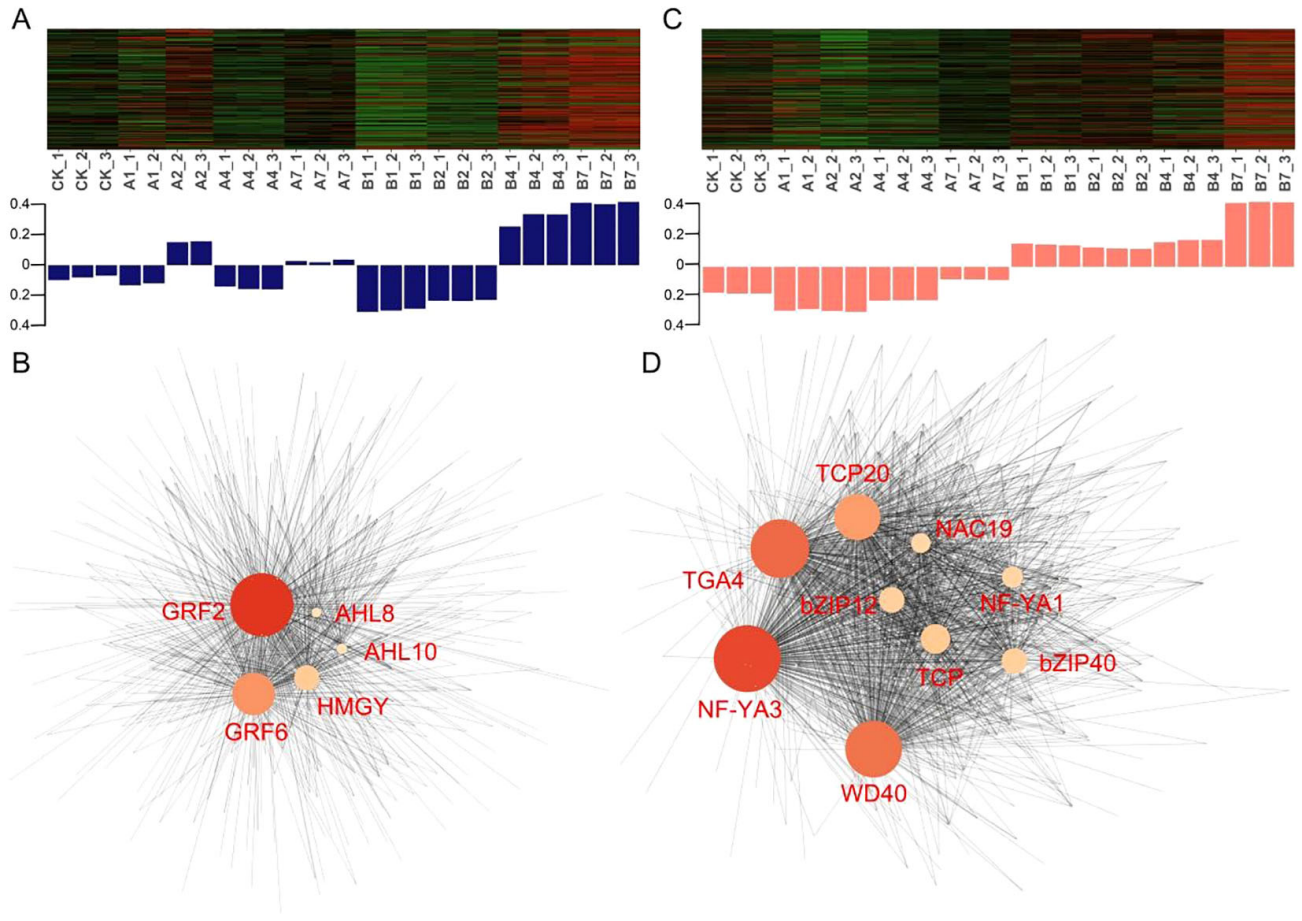
Expression profile and correlation network of genes within two modules. Expression profile of genes in nine samples of midnightblue module **(A)** and salmon module **(C)**. **(A, B)** represent the seedlings of *B. laciniata* treated with 20% and 30% PEG, respectively. CK represents 0 day post-PEG treatment. 1, 2, 4, 7 represent the days after PEG treatment. The bar-plot reported eigengene expression at each sampling point. The y-axis indicates the eigengene expression value. Network construction of hub genes in midnightblue module **(B)** and salmon module **(D)**, TFs were shown in red font.

Additional examination of gene expression patterns in the midnightblue and salmon modules shows that plants exposed to 30% PEG exhibited higher expression levels compared to those treated with 20% PEG (**Figures 5A, C**). Using the Hub–TFs and their correlation network (**Supplementary Table S6**), we built and visualized a network closely linked to drought stress, applying a weight threshold greater than 0.2 (**Figures 5B, D**). Overall, 5 transcription factors were found in the midnightblue module and 9 in the salmon module (**Figures 4B, D**, **Supplementary Table S7**). Finally, GRF2 and NF-YA3, originating from the midnightblue and salmon modules respectively, were recognized as potential transcription factors for drought resistance in *B. laciniata*, as they occupied central positions in the gene regulatory network. The RT-qPCR result indicated that these two genes were induced by PEG stress (**Supplementary Figure S3**).

## Discussion

The effects of drought stress on plant yield and quality are exacerbated by global warming and increasing water shortages (Lesk et al., 2016; Gupta et al., 2020; Easterling et al., 2000; Su et al., 2018). While the overarching impacts of drought stress on plant phenotypic development are extensively documented in the literature, the precise mechanistic underpinnings at biochemical cascades and molecular regulatory networks remain insufficiently elucidated. Therefore, investigating the mechanisms underlying plant drought resistance and identifying key drought resistance genes are crucial for ensuring the sustainable development of the plant industry (Raposo et al., 2023). An increasing body of research highlights the value of medicinal plants as resources for stress resistance (Ye et al., 2025; Vaghela and Gohel, 2023; Tan and Gören, 2024). Notably, in this study, *B. laciniata*, a traditional herbal medicine, has demonstrated tolerance to drought stress in natural environments (**Supplementary Figure S1**). Understanding plant responses to adverse stress is essential for enhancing agricultural productivity through innovative farming practices and plant breeding (Rivero et al., 2022). Consequently, the primary research objective of this paper is to elucidate the molecular mechanisms of drought resistance in *B. laciniata*, thereby providing a theoretical foundation for molecular breeding.

When plants encounter environmental stresses, the most immediate and observable effect is growth inhibition. Following drought stress, plants undergo a series of morphological and physiological changes to adapt to external stress. Under simulated drought stress induced by PEG, both 20% and 30% concentrations resulting in gradual chlorosis, marginal curling, wilting, and sporadic necrotic lesion formation, with 30% PEG showing greater damage (**Figures 1A, B**). Pro acts as an osmolyte, ROS scavenger, and molecular chaperone for stabilizing protein structures (Szabados and Savouré, 2010). In addition, drought stress can cause increased activity of enzymes such as SOD (Mishra et al., 2011). We only measure two these indicators, which has certain limitations, but our investigation demonstrated a progressive elevation in both pro content and SOD activity within *B. laciniata* seedlings subjected to drought stress, with 30% PEG showing greater increasing (**Figures 1C, D**).

In response to the detrimental effects of drought, plants have evolved a range of strategies that involve reconfiguring their transcriptome, proteome, and metabolome profiles to increase their resilience to stress (Liu et al., 2023; Ilyas et al., 2021; Martin‐StPaul et al., 2017, Zhu and JK, 2016). Moreover, using genome-wide transcriptome analysis methods offers comprehensive insights into gene regulatory networks and aids in identifying stress-responsive genes in plants (Abdel-Ghany et al., 2020; Ye et al., 2025). Therefore, this study reveals the gene expression changes of *B. laciniata* under different PEG concentration induction stress through transcriptome analysis. A total of 57,502 genes were identified (**Supplementary Table S1**). The PCA results showed that the biological replicates were clustered together, indicating that the transcriptome data were reliable and repeatable (**Figure 2A**). The transcriptome profile of nine samples from each time point was compared to comprehend the difference in expression profile, and 39,215 DEGs in at least one combination were identified (**Supplementary Table S2**). The number of DEGs in the eight comparisons (A1 vs. CK, A2 vs. A1, A4 vs. A2, A7 vs. A4, B1 vs. CK, B2 vs. B1, B4 vs. B2, and B7 vs. B4) ranges from 7,399 to 19,507 (**Figure 2B**). These results demonstrate distinct transcriptional reprogramming in *B. laciniata* seedling under drought stress, consistent with previous studies (Abdel-Ghany et al., 2020; Ye et al., 2025).

Venn analysis revealed 825 common DEGs in the comparisons of A1 vs. CK, A2 vs. A1, A4 vs. A2, and A7 vs. A4, and 1450 common DEGs in the comparisons of B1 vs. CK, B2 vs. B1, B4 vs. B2, and B7 vs. B4 (**Figures 3A, B**). These common gens underwent a comprehensive KEGG pathway enrichment analysis. Previous studies indicated that expression patterns of multiple genes related to various biological pathways, including phenylpropanoid biosynthesis and flavonoid biosynthesis, are altered in response to drought stress by trancripomics analysis (Bashir et al., 2019; Vogt, 2010; Feng et al., 2024). In line with earlier research, our findings revealed that both phenylpropanoid biosynthesis and flavonoid biosynthesis pathways were enriched in the two common gene sets (**Figures 3C, D**), suggesting that *B. laciniata* reacts to drought stress by modulating phenylpropanoid and flavonoid pathways.

WGCNA is a novel gene screening method that can be used to explore relationships between traits and expression profiles (Chen et al., 2024b; Yang et al., 2023). Through WGCNA analysis in this study, the midnightblue and salmon modules were found to be strongly linked to pro and SOD, respectively (**Figure 4**), with 472 hub genes identified across the two modules (**Supplementary Table S5**). Transcription factors serve as crucial regulators, managing gene expression for various biological and metabolic processes during drought stress (Manna et al., 2021; Zhang et al., 2023; Tang and Xia, 2025). Understanding how transcription factors function and are regulated will help in crafting crop improvement strategies to create and distribute crops with better agronomic qualities. Therefore, through network analysis, we identified 5 and 9 TFs in midnightblue and salmon, respectively (**Figures 5B, D**, **Supplementary Table S7**). Specifically, GRF2 from the midnightblue module and NF-YA3 from the salmon module were chosen as potential transcription factors for regulating the resistance of *B. laciniata* to drought stress. According to the RT-qPCR results, PEG stress led to the activation of these two genes (**Supplementary Figure S3**). GRFs and NF-Ys play important roles in regulating abiotic stress response (Cui et al., 2024; Feng et al., 2015). Overexpressing *AtGRF7* causes *DREB2A* to be up-regulated, which boosts resistance to salt and drought conditions (Sakuma et al., 2006). Additionally, drought tolerance was observed in Arabidopsis that overexpressed wheat NF-YA10-1 (Ma et al., 2015). These papers back up our hypothesis that GRF2 and NF-YA3 may be involved in drought resistance. The findings from the transcriptomic analysis in this research shed light on the mechanisms behind the response of *B. laciniata* to drought stress.

## Data Availability

RNA sequence data were deposited in the NCBI with BioProject accession number PRJNA1357639.
