# Supplementary material for: Transcriptomic analysis of Blumea laciniata responses to PEG-simulated drought stress
Source: Front Plant Sci. 2025 Dec 18;16:1695003. doi: 10.3389/fpls.2025.1695003 (PMC12756431; doi:10.3389/fpls.2025.1695003)
Supplement: Supplementary file 1 [file DataSheet1.docx]

**Supplementary Materials**


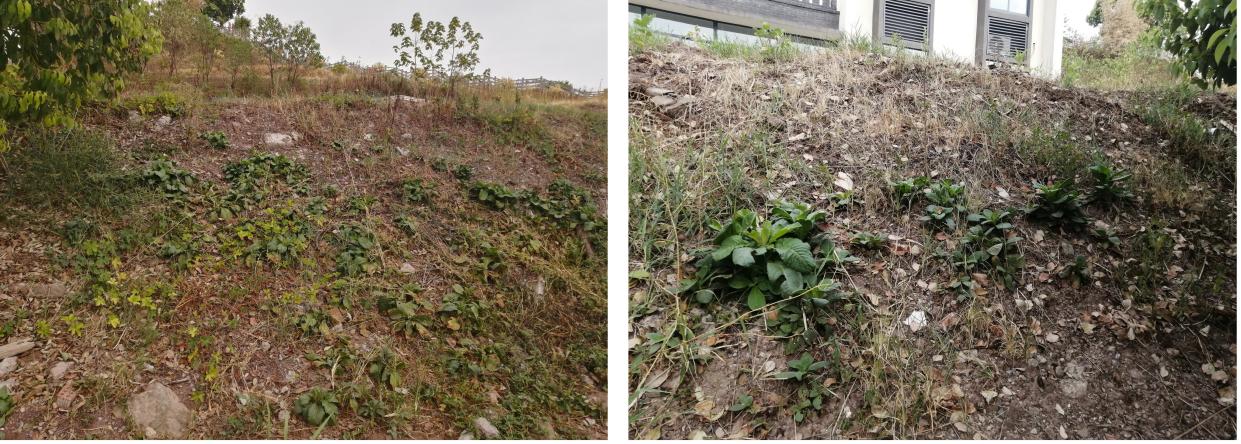


**Figure S1.** Phenotypic characteristics of *B. laciniata*. The left panel's plants were photographed on August 26, 2023, and the right panel's plants on August 28, 2023, with all photos taken in Baishiyi Town, Jiulongpo district, Chongqing.


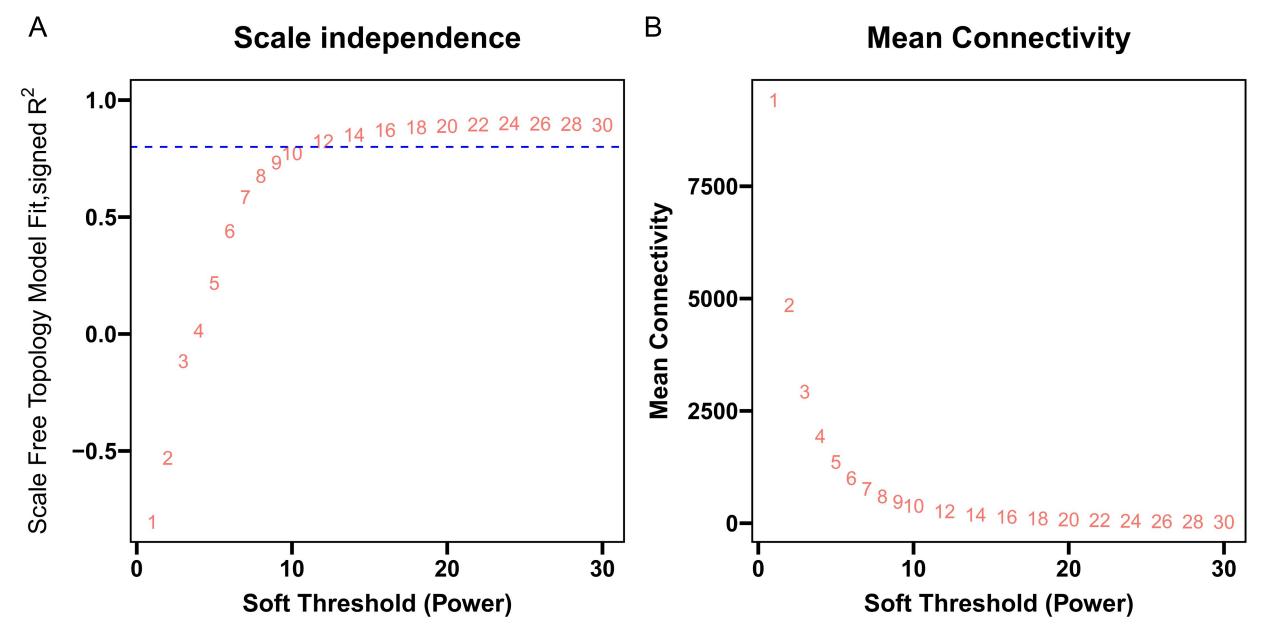


**Figure S2. Weighted-gene co-expression network. (A)** Scale free topology model fit. The dashed line represents R^2^ = 0.8. (B) Mean connectivity.


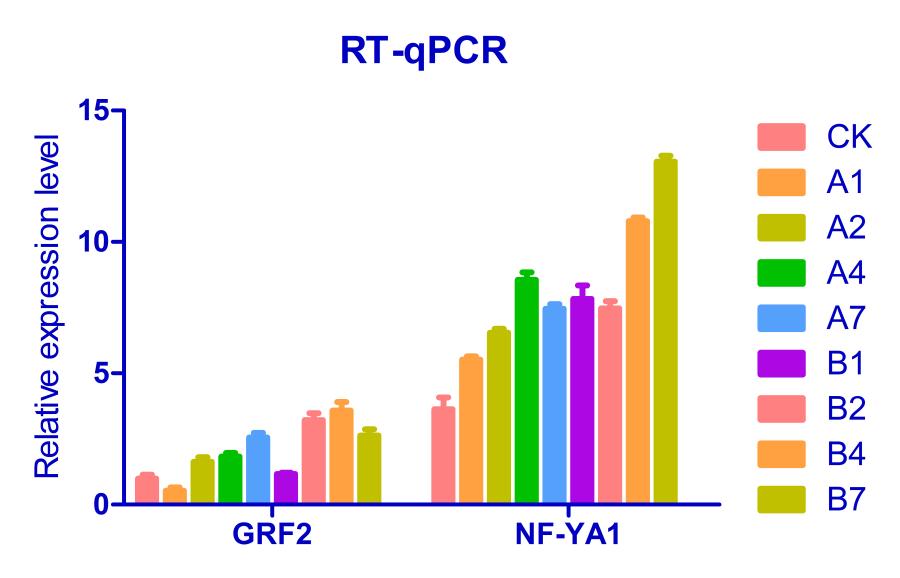


**Figure S3. The expression level of *GRF2* and *NF-YA1* in leaves of *B. laciniata* analyzed by RT-qPCR.** Total RNAs were extracted from leaves of *B. laciniata*. Actin (g16517) was used as the reference gene for normalization. Three biological replicates were performed. Figures were drew with mean ± SD. A and B represent the seedlings of *B. laciniata* treated with 20% and 30% PEG, respectively. CK represents 0 day post-PEG treatment. 1, 2, 4, 7 represent the days after PEG treatment.
